# Institutional Housekeeping

**Published:** 1911-10-28

**Authors:** 


### INSTITUTIONAL HOUSEKEEPING.

(Contributions invited: Workers' Questions will be welcome.)

### The Treatment of Tough Meat.

Every housekeeper and cook has at some time or another experienced acute vexation on hearing the meat provided for dinner or supper described as "tough as leather," the inevitable results being complaints from the consumers and either waste of material or attacks of indigestion, with all their various and injurious symptoms.

First of all, to understand *why* meat should toughen, it is necessary to consider as simply as possible the structure of flesh. What we term the "lean meat" of animals consists of little bundles of muscular tissue, bound and held together by thin, tough sheets of membrane, called connective tissue. If these bundles were examined under a microscope, they would be found to be made up of very fine muscular fibres held by still finer and thinner membranes than the outer bundles. What we want to note here is the presence of fibres and thin membranes, and that the latter are largely composed of a substance which when boiled produces gelatine. Then comes the keynote of the whole matter, namely, that muscular fibres contain an important egg-like substance called albumen. This is distributed all through the meat, and heat has just the same action on it as it has on white of egg. First it becomes opaque, and semi-coagulated; then if subjected to greater heat it is quickly converted into a hard, tough mass. Thus it can be easily understood that if meat is heated to such a degree that the albumen it contains passes the soft, opaque stage and becomes tough, it will be no more easily masticated or digested than the white of an egg boiled for about half an hour. In fact it will be worse than the white of egg, for intermingling with the coagulated albumen will be stringy fibres.

There are two forms under which tough meat is encountered.

The tough, leathery joint or steak, hardened, shrivelled, distorted and curled up, with an india-rubber-like texture, has simply been boiled sufficiently long to harden the albumen and contract the muscular fibres.

The apparently tender piece of meat, which leads the inexperienced to hope great things, is of another origin. This fictitiously tender meat falls to pieces when cut and yet proves practically tough and stubbornly resistant when the teeth endeavour to masticate the separated fibres.

What has occurred is that the cook has simmered the meat at too high a temperature, until she has succeeded in converting it into a leathery mass, and then, "to make it tender," has continued the so-called simmering until the gelatine of which the connective tissues or membrane are composed has, after the nature of gelatine, partially or entirely dissolved, thus permitting the already toughened fibres to become easily separable and the flavour and the nutritive value of the solid part to be lost. Having already grasped the fact that the kitchen is mainly responsible for tough meat, the next step is to find out how best technical errors on the cook's part can be avoided. Evidently the degree of heat to which the meat is to be subjected is the main point, and here is the rock on which the health of thousands is wrecked.

Boiled or stewed meat is cooked in either water or weak stock, and the boiling point of these is 212° Fahr. As meat contains albumen, and as this substance can be exuded from the meat and diffused in cold water, which would obviously often mean its entire loss, it is frequently necessary to seal up and harden the outer layers of meat by the application of sharp heat in order that the nutritive juices may not be drawn out and wasted. To ensure this the water must be at boiling point.

If, however, this temperature is kept up for longer than five to ten minutes, according to the thickness of the meat, the albumen will harden all through the meat, with the result already described. From this is learnt:—

(1) The need of first plunging meat into boiling water to seal the outside, unless it is desired to extract some of its juices into the gravy, when cold water is used.

(2) After the first 5 to 10 minutes to lessen the heat to 160° to 180° Fahr. Bubbling must cease, or just one or two bubbles may appear at the side of the pan nearest the hottest part of the fire. Cooking thermometers are so inexpensive and simple that the merest culinary novice can use them and no institution kitchen should be without one.

Some parts of meat require to be kept only a degree or two below boiling point during the cooking process. Some of these are: Shin of beef, cow-heel, sheep's trotters, pigs' feet, knuckles of veal, and parts consisting mainly of gristly tendons and gelatinous substances.

With a little experience those in charge of the catering department can soon detect meat that will probably be tough when cooked:—

(1) Meat that is freshly killed, such as is often delivered in hot weather, when hanging meat is impossible.

(2) Meat of poor quality, with coarse, stringy, loose fibres showing along the cut surfaces.

(3) Meat of old animals, distinguishable by the yellow horny strip of gristle and hard muscles.

(4) The cheaper, coarser parts of meat, such as scrag end of neck of mutton.

Any of the above will prove tough and unsuitable for roasting, grilling, or frying.

The greater the likelihood of meat proving tough the slower and longer must be the process of cooking. If the fibres are coarse, soak it for a few minutes in vinegar, as the action of the acid aids in softening the fibres.

To safeguard against hardening meat by boiling, a double pan can be used, the outer vessel holding the water which is kept at simmering point, and the smaller inner one containing the meat, liquid, etc.

